# Polypseudophakia with the J&J synergy lens in high myopia: case report

**DOI:** 10.1097/RC9.0000000000001076

**Published:** 2026-10-14

**Authors:** David Durgan, Noah Collins, Jessica Kim, Frank Hwang

**Affiliations:** a Department of Ophthalmology, Loma Linda University School of Medicine, Loma Linda, CA, USA; b Department of Anesthesiology and Pain Medicine, University of Washington, Seattle, WA, USA; c Department of Ophthalmology, Loma Linda University Eye Institute, Loma Linda, CA, USA

**Keywords:** case report, high myopia, multifocal, polypseudophakia, Uveitis-Glaucoma-Hyphema syndrome

## Abstract

**Introduction and importance:**

As clinicians strive to provide personalized recommendations for patients, polypseudophakia may be an option for those outside the range of traditional extended depth-of-focus lenses. We describe a case with initial refractive success but the development of a potentially vision-threatening complication.

**Presentation of case:**

A 40-year-old Asian man with high myopia presented for a refractive surgery consultation with a goal of total spectacle independence and underwent bilateral refractive lens exchange with the J&J Synergy lens in the capsular bag, and a minus-power Alcon MA60MA lens in the sulcus. Initially, there was refractive success with post-operative uncorrected distance vision of 20/20 in the right eye and 20/25 in the left eye at 6 months. However, both eyes developed transillumination defects with pigment in the anterior chamber, consistent with Uveitis-Glaucoma-Hyphema (UGH) syndrome. The patient ultimately elected bilateral explantation of the sulcus intraocular lenses (IOLs) due to this complication.

**Clinical discussion:**

To our knowledge, this is the first case using the J&J Synergy lens in polypseudophakia and the second to correct for high myopia. Surgical techniques for those with high myopia and thin corneas are rare, and this technique initially provided a hopeful refractive outlook. However, loss of the refractive target and the development of UGH syndrome challenged the safety of this technique.

**Conclusion:**

Further studies are needed to determine which patients can tolerate this procedure. Providers should proceed cautiously and anticipate complications such as UGH syndrome following secondary sulcus IOL placement.

## Introduction

The history of polypseudophakia dates back to 1993, when Gayton and Sanders published a case of the implantation of two posterior chamber intraocular lenses (IOLs) for the treatment of microphthalmos^[^1^]^. The technique has become more popular, with either both lenses being placed simultaneously to correct severe hyperopia or a second lens being placed later in cases of refractive surprise. In 2007, Akaishi *et al* published a series of 20 eyes that underwent IOL implantation with a Tecnis ZM900 multifocal IOL and a second silicone IOL in the sulcus^[^2^]^. Akaishi also published a series of 13 eyes that underwent IOL implantation with the AcrySof ReSTOR lens and a silicone 3-piece IOL in the sulcus^[^3^]^. While several other cases have been described, we found only one case in which this technique was used to correct high myopia^[^4^]^. To our knowledge, this is the first case of polypseudophakia involving the J&J Synergy lens, and the second case using the polypseudophakia lens system to correct high myopia. This case report has been prepared in line with the SCARE checklist^[^5^]^.

HIGHLIGHTS

- Use of secondary sulcus IOL placement in high myopia.
- Development and management of Uveitis-Glaucoma-Hyphema syndrome.
- First implementation of the J&J Synergy in a two-lens system.
- Discussion of pre-operative considerations for sulcus lens placement.

## Case presentation

A 40-year-old Asian man with a history of bilateral high myopia presented to an academic medical center for a refractive surgery evaluation, with a goal of total spectacle independence through a single surgical option. Past ocular history was notable for myopic degeneration and a single episode of iritis in the right eye 1 year prior to the surgical evaluation. This episode was thought to be related to contact lens overwear and resolved with topical steroids. No uveitis workup was performed. His manifest refraction at the time of presentation was −17.00 sphere in the right eye and −19.00 + 1.00 × 045 in the left eye, which had been stable over the past year. Best corrected visual acuity (BCVA) was 20/40 in the right eye and 20/50 in the left eye, limited by mild myopic degeneration. Pachymetry was 506 and 492 µm in the right and left eyes, respectively. The calculated ablation would have left 166 µm in the right eye and 112 µm in the left eye of residual stromal bed (RSB). The minimum safe RSB is considered to be 250 µm; however, most conservative surgeons prefer to leave 300 µm. Due to inadequate corneal thickness with high myopia, the decision was made to proceed with refractive lens exchange instead of laser refractive surgery. Preoperative examination included a Pentacam (Fig. 1A–D). IOL measurements were taken using the IOL Master 700 (Fig. 1E). Additional pre-operative patient measurements are listed in Table 1. The angles were open, and the eyes were quiet at the time of surgery. No pre-operative findings were suggestive of future iris-lens contact.

**Figure 1.**
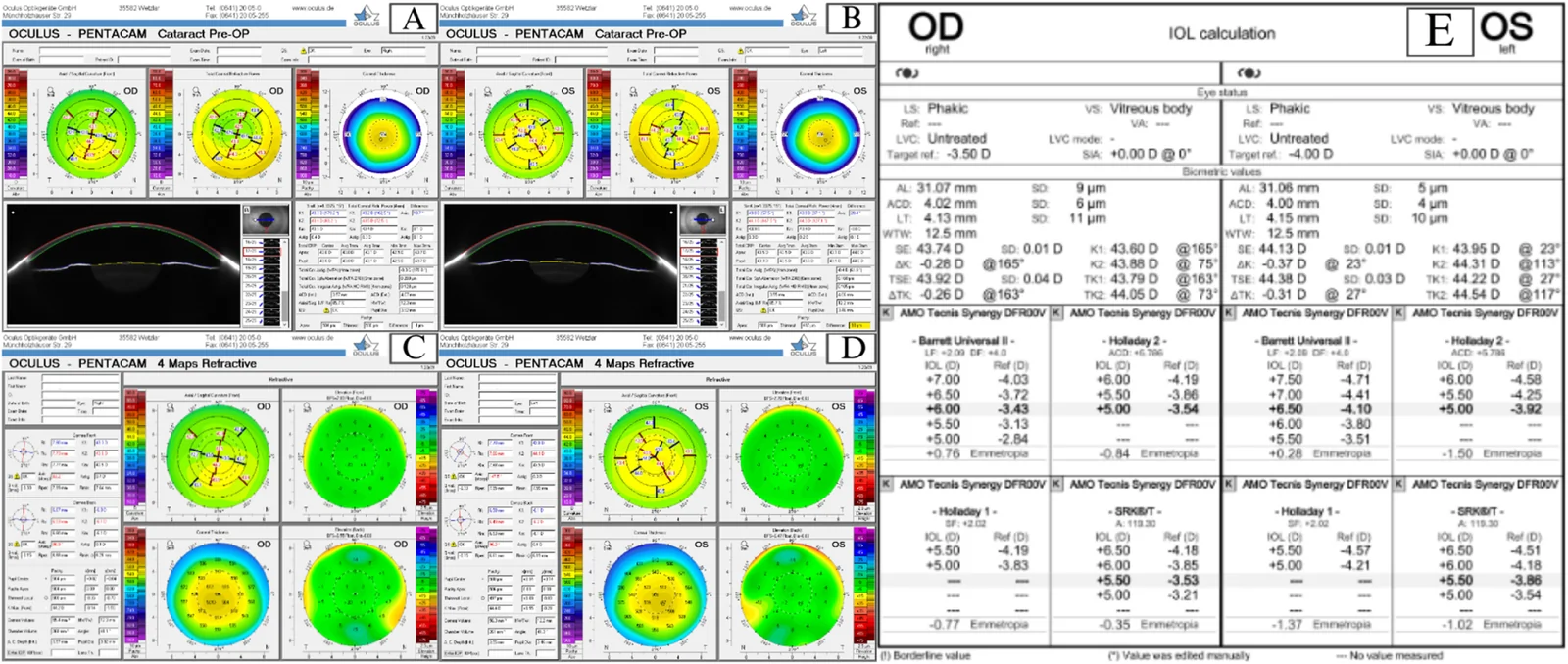
(A) Pentacam preoperative examination of the right eye showing the angle between the cornea and the iris. (B) Pentacam preoperative examination of the left eye showing the angle between the cornea and the iris. (C) 4 Maps Refractive Pentacam, preoperatively, in the right eye. (D) 4 Maps Refractive Pentacam, preoperatively, in the left eye. (E) IOL power predictions for the right and left eyes using the IOL Master 700, including axial length, corneal power, and IOL calculations (Barrett Universal II, Holladay 2, Holladay 1, SRK/T) with the AMO Tecnis Synergy DFR00V lens.

**Table 1:** Shown are the patient’s pre-operative measurements.

	Manifest refraction	Pre-op BCVA	K1	K2	Axis	Axial length (mm)	Corneal thickness (microns)	ACD (mm)
OD	−17.00 DS	20/40	43.3	43.6	83.2	31.07	506	4.02
OS	−19.00 + 1.00 × 045	20/50	43.8	44.1	147.5°	31.06	492	4.00

ACD, *=* Anterior Chamber Depth; BCVA, Best Corrected Visual Acuity; DS, Diopter Sphere; K1, Flat Keratometry; K2, Steep Keratometry; OD, Right Eye; OS, Left Eye.

The patient underwent an uncomplicated left eye refractive lens exchange. The surgeon was a fellowship-trained ophthalmologist in cornea and external disease with over 15 years of relevant experience. A J&J Synergy 5.0 D lens was selected because it was the lowest-power multifocal or extended depth-of-focus lens available at the time, which would leave the patient with the least refractive error in case the sulcus lens needed to be removed later. This was placed in the capsular bag, followed by a −4.0 D Alcon MA60MA in the sulcus after confirmation with the Optiwave Refractive Analysis (ORA) System. During post-operative week 1, uncorrected distance visual acuity (UDVA) was 20/20 with J3 at near. Routine post-operative drops, including nonsteroidal anti-inflammatory and steroid drops, were tapered throughout the first month. At post-operative month 6, UDVA was 20/25.

Given the stability and excellent refractive outcome, the same surgical technique was applied in the right eye 1 month later, with a −3.0 D Alcon MA60MA lens in the sulcus. Due to a prior history of iritis in the right eye, the patient received a Medrol dose pack post-operatively. The patient was followed by the same surgeon at the same academic medical center. During post-operative week 2, UDVA was 20/25 and J2 at near. However, the patient had an increased anterior chamber reaction and was started on prednisolone drops. At post-operative month 1, UDVA was 20/20. At post-operative month 6, though inflammation improved, he had pigment dispersion (Fig. 2A) and iris transillumination defects consistent with Uveitis-Glaucoma-Hyphema (UGH) syndrome (Fig. 2B). Ultrasound biomicroscopy (UBM) showed iris-lens touch in the right eye (Fig. 3). At this visit, UDVA was 20/20 in the right eye. The patient was pleased with his UDVA after surgery. He was counseled on the presence of persistent pigment dispersion, iris-lens touch in both eyes, and the risk of glaucoma. The patient ultimately chose explantation of the sulcus lens in both eyes for definitive treatment. He planned for refractive correction with glasses and soft contact lenses, with the option of laser vision correction for residual refractive error in the future. Three months after explantation, the patient achieved a BCVA of 20/20 in the right eye and 20/40 in the left eye, which was limited due to mild myopic macular degeneration. Vision remained the same 1.5 years after explantation. At 3.5 years following explantation, at the most recent follow-up, vision was 20/20 in the right eye and 20/100 in the left, due to myopic degeneration that had progressed more in the left than the right eye. Intraocular pressure was 15 in both eyes. There has been no recurrence of intraocular pressure increase or iritis since explantation.

**Figure 2.**
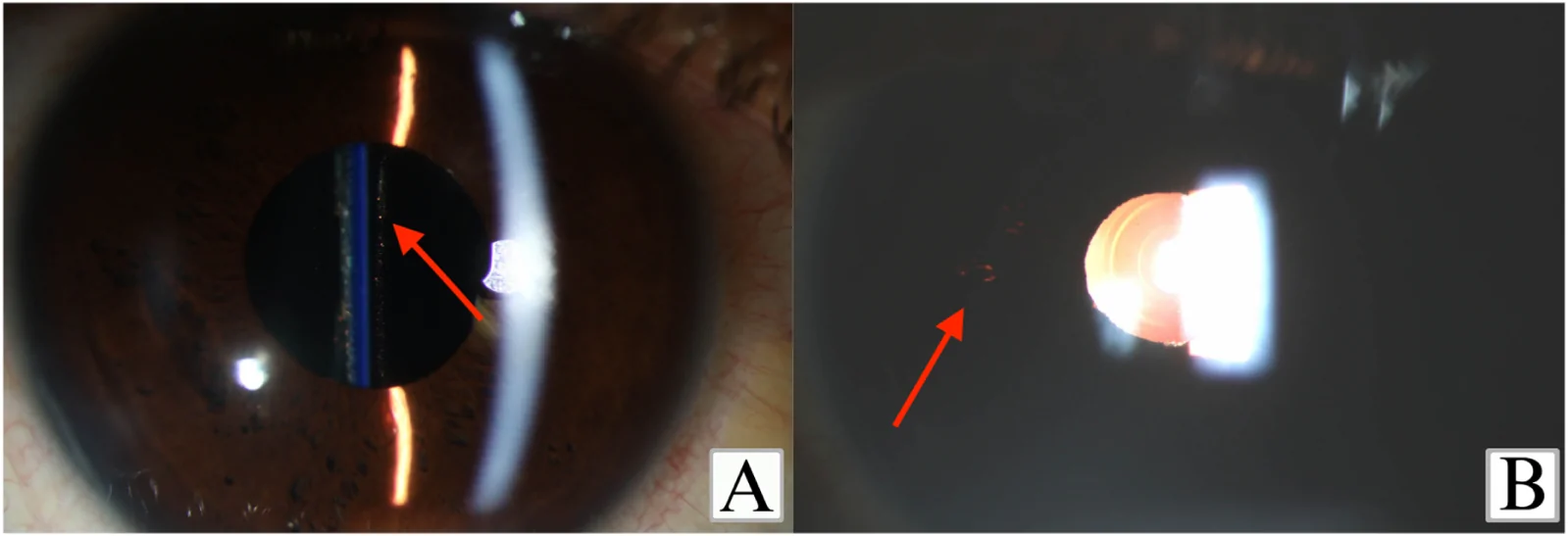
(A) Slit-lamp photograph of the right eye showing dispersed pigment on the iintraocular lens (red arrows). (B) Slit-lamp photograph of the right eye showing transillumination defects of the iris (red arrows).

**Figure 3.**
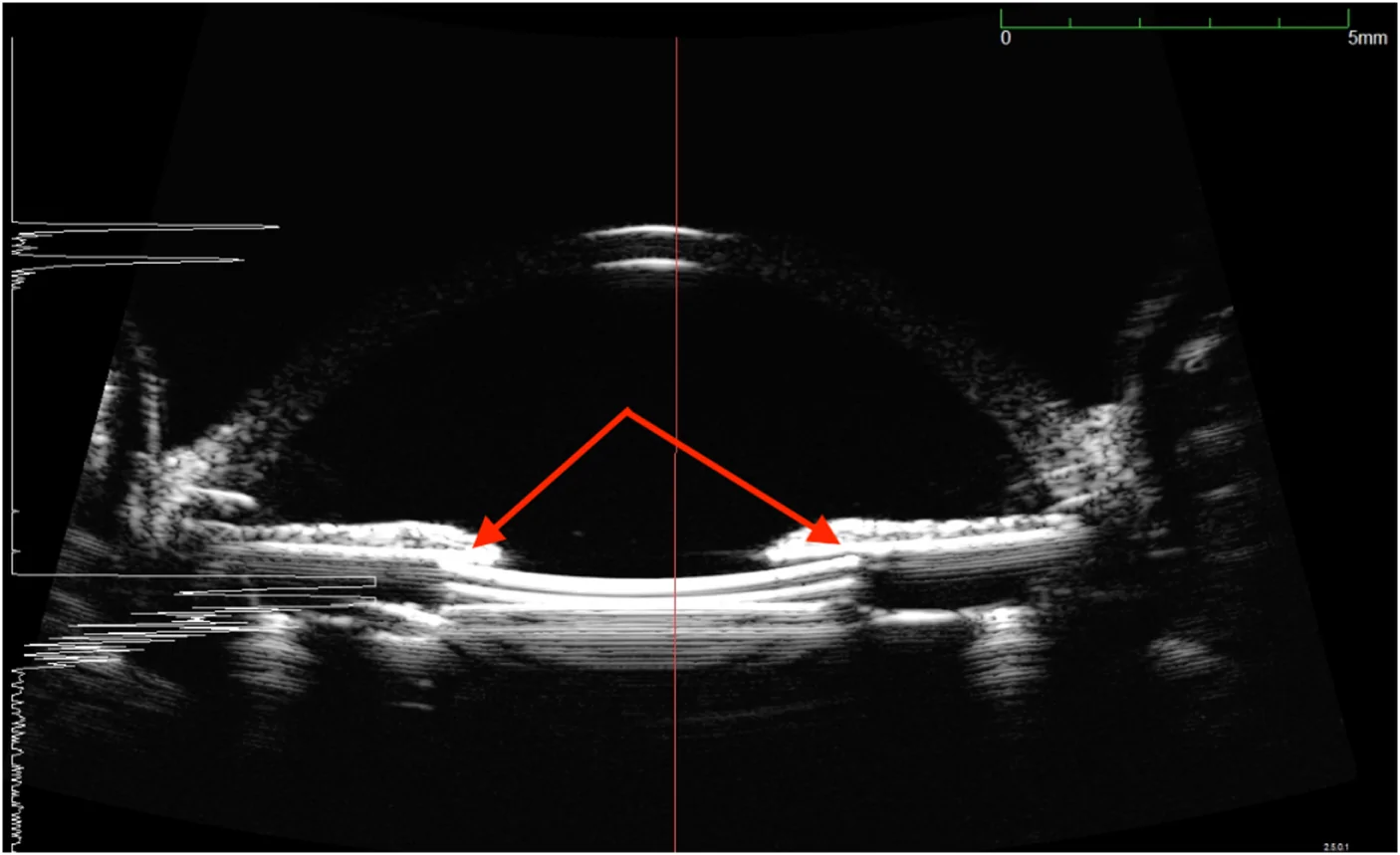
Post-operative ultrasound biomicroscopy (UBM) of the right eye showing iris-intraocular lens (IOL) touch (indicated by red arrows). Post-operative UBM is unavailable for the left eye.

## Discussion

With growing technological advancements in refractive surgery, there is an increased drive to provide patients with personalized solutions. Previously, in young patients with very high myopia and thin corneas, it has been difficult to provide refractive correction for distance without compromising near vision, as low-powered IOLs are not available in the U.S. to correct the refractive error as a standalone treatment. The advent of secondary sulcus lenses has increased possibilities, but they have mainly been used for hyperopes.

After extensive review and discussion with manufacturers, it was determined that a multifocal minus-powered lens suitable for our needs was unavailable in the U.S. market (Zeiss AT LISA). Additionally, the manufacturer confirmed that there are no plans to introduce such products in the future. Our lens choices were further limited by COVID-19-related supply chain issues, leaving us with the J&J Synergy, which combines extended depth-of-focus and multifocal technologies to provide a range of vision. Our patient was a poor candidate for keratorefractive surgery due to the need for high ablation depths to treat his high myopia on relatively thin corneas. Also, he wished to pursue a single surgical option. After multiple extensive discussions, we considered our surgical plan to be the best option for him, since he desired functional near vision. Previously, this technique had been used in myopia without complications and with excellent refractive results^[^4^]^. While the technique had a great initial refractive outcome, it resulted in UGH syndrome, challenging the safety of this method. This complication is less likely to have developed solely as idiopathic inflammation because of the patient’s remote history of iritis, though this may have played a role in the early inflammation. While there is concern for UGH syndrome with the placement of a lens in the sulcus, UBM was not used preoperatively, which was a limitation of the surgical planning process. We contemplated placing both IOLs in the bag but ultimately did not due to concern for interlenticular opacification^[^6^]^.

Our patient ultimately elected bilateral explantation of the sulcus lenses due to the development of UGH syndrome, with subsequent loss of the refractive goal. The development of UGH syndrome was likely due to mechanical friction between the iris and the Alcon MA60MA lens in the sulcus, which raises concerns about the safety of this lens combination. This lens is designed for implantation in the capsular bag rather than in the sulcus. It has a squared optic edge and a meniscus optic design. Lenses designed for sulcus-specific IOL implantation, such as the Rayner Sulcoflex IOL, have a rounded optic edge and a posterior concave surface designed to reduce iris chafing and the likelihood of UGH syndrome. This lens may have been beneficial in this case, but unfortunately, such sulcus-specific IOLs are not widely available for use in the United States.

There are previous reports of using Laser Peripheral Iridotomy (LPI) to treat cases of UGH syndrome from reverse pupillary block. However, the patient did not exhibit any of the clinically characteristic signs of reverse pupillary block on examination, which include posterior bowing of the iris on slit-lamp examination or UBM, or the “Finger of God” sign^[^7^]^. Even though LPI and medical management of UGH syndrome with long-term topical drops were considered, neither addressed the underlying mechanical etiology. LPI would not have alleviated the iris-lens touch, because the UGH syndrome was thought to be from mechanical friction rather than reverse pupillary block. Topical drops could decrease intraocular pressure, but they would require lifetime use and monitoring, creating a significant treatment burden. Through shared clinical decision-making with the patient, the decision was made to explant the sulcus lens bilaterally for definitive treatment. Previously, high myopia has been suggested as a major risk factor for pigment dispersion and UGH syndrome following the sulcus placement of posterior chamber intraocular lenses^[^7^]^. We suggest that clinicians consider increased axial length as a risk factor for developing UGH syndrome and recommend greater caution with implantation of sulcus IOLs in these eyes.

The limitations of this study include the lack of pre-operative UBM in the surgical planning process and the inability to obtain a narrative patient perspective despite multiple attempts. Additionally, post-operative UBM was not obtained for the left eye. The same etiology was inferred for the left eye based on similar clinical findings, but without objective confirmation. Finally, as a single case report, generalizability to the general population is limited.

Providers should use caution when attempting to use this technique and consider routinely including UBM as part of the pre-operative examination to prevent complications from iris-lens touch. Additionally, when possible, sulcus-designed lenses with rounded optic edges should be used rather than lenses with squared optic edges. Patients with squared optic edges lenses in the sulcus should be followed closely to monitor for complications. Further studies are needed to evaluate the long-term outcomes and safety of this technique in myopic patients and to determine which patients may be suitable candidates.

## Data Availability

Not applicable.

## Prior presentation

The surgical video for this case was previously presented at the Canadian Ophthalmology Society Annual Meeting in 2022.
